# Case Report: Periungual Xeligekimab injection for refractory Acrodermatitis continua of Hallopeau

**DOI:** 10.3389/fimmu.2026.1753175

**Published:** 2026-03-03

**Authors:** Peng Cao, Aijie Yuan, Jingchen Yang, Yuning Zhang, Tao Guo, Chen Li

**Affiliations:** 1 Department of Dermatology, Tianjin Academy of Traditional Chinese Medicine Affiliated Hospital, Tianjin, China; 2 Graduate School, Tianjin University of Traditional Chinese Medicine, Tianjin, China; 3 School of Nursing, Beijing University of Chinese Medicine, Beijing, China; 4 Department of Dermatology, Tianjin Institute of Integrative Dermatology, Tianjin Academy of Traditional Chinese Medicine Affiliated Hospital, Tianjin, China

**Keywords:** acrodermatitis continua of Hallopeau, case report, IL-17, periungual injection, xeligekimab

## Abstract

This case report presents the novel and successful use of periungual Xeligekimab injections for managing refractory Acrodermatitis Continua of Hallopeau (ACH), representing the first documented application of this localized administration route for the IL-17A inhibitor. It contributes to the scientific literature by demonstrating a promising alternative therapeutic strategy for treatment-resistant ACH, highlighting the potential for enhanced local efficacy and minimized systemic exposure. The patient was a 36-year-old woman with a two-decade history of ACH affecting her digits, characterized by persistent periungual and subungual erythema, sterile pustules, significant nail plate dystrophy with thickening and fragmentation, and associated digital swelling, tenderness, and restricted motion. Prior therapies, including topical corticosteroids, phototherapy, and systemic tofacitinib, had proven ineffective. Diagnosis was confirmed clinically and supported by MRI, which revealed active bone marrow edema in the distal phalanges. The therapeutic intervention involved initial periungual injections of a diluted Xeligekimab formulation, which led to partial improvement. This was followed by a series of injections using undiluted Xeligekimab (100 mg/mL). This escalation resulted in marked clinical resolution: complete clearance of pustules and inflammation, healthy nail regrowth, and resolution of digital swelling and pain. Concurrently, the Dermatology Life Quality Index and pain scores dramatically improved, and follow-up MRI showed substantial resolution of the underlying bone marrow edema, confirming the treatment’s efficacy on both cutaneous and deep osseous inflammation.

## Introduction

Acrodermatitis continua of Hallopeau (ACH) is a rare, chronic variant of pustular psoriasis. The disease is characterized by the development of sterile pustules around and beneath the nail, periungual inflammation, nail dystrophy, osteolysis of the distal phalanges, and progressive pain and atrophy of the affected digits. This localized form of pustular psoriasis predominantly affects the distal portions of the fingers and toes (1). Current management strategies usually follow the international treatment guidelines for psoriasis and include the administration of topical calcineurin inhibitors, phototherapy, and systemic agents such as methotrexate. In recent years, biologic therapies targeting the interleukin-17 (IL-17), IL-23, and IL-36 signaling pathways have shown significant efficacy in the treatment of refractory ACH (2, 3). Here, we report a case of treatment-resistant ACH that was successfully managed using an innovative approach, periungual injection of Xeligekimab, resulting in substantial clinical improvement of the digital lesions.

## Case presentation

A 36-year-old woman presented with a 20-year history of recurrent erythema and sterile pustules around the nail folds of the left ring finger and right thumb, which had progressed gradually to thickening, opacification, and fragmentation of the nail plate. Over the past year, she had developed persistent swelling, tenderness, and restricted motion of the affected digits. The patient had previously received prolonged topical corticosteroid therapy and phototherapy without lasting improvement. Treatment with the Janus kinase (JAK) inhibitor tofacitinib for six months at another institution had failed to control the disease, with worsening of the symptoms. She denied any personal or family history of psoriasis or autoimmune disorders.

**Figure 1:**
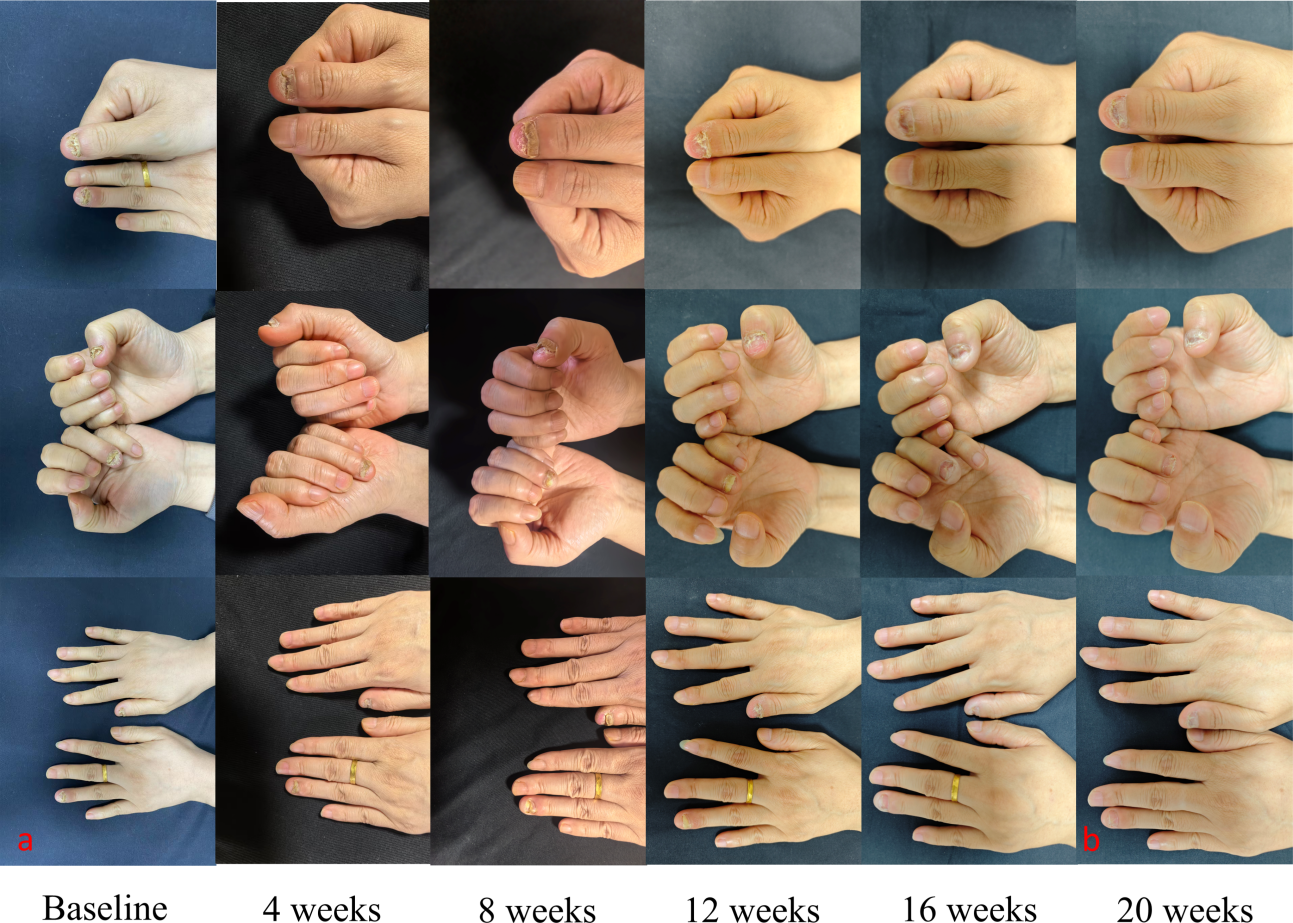
Clinical presentation at baseline and after treatment with Xeligekimab.

**Figure 2:**
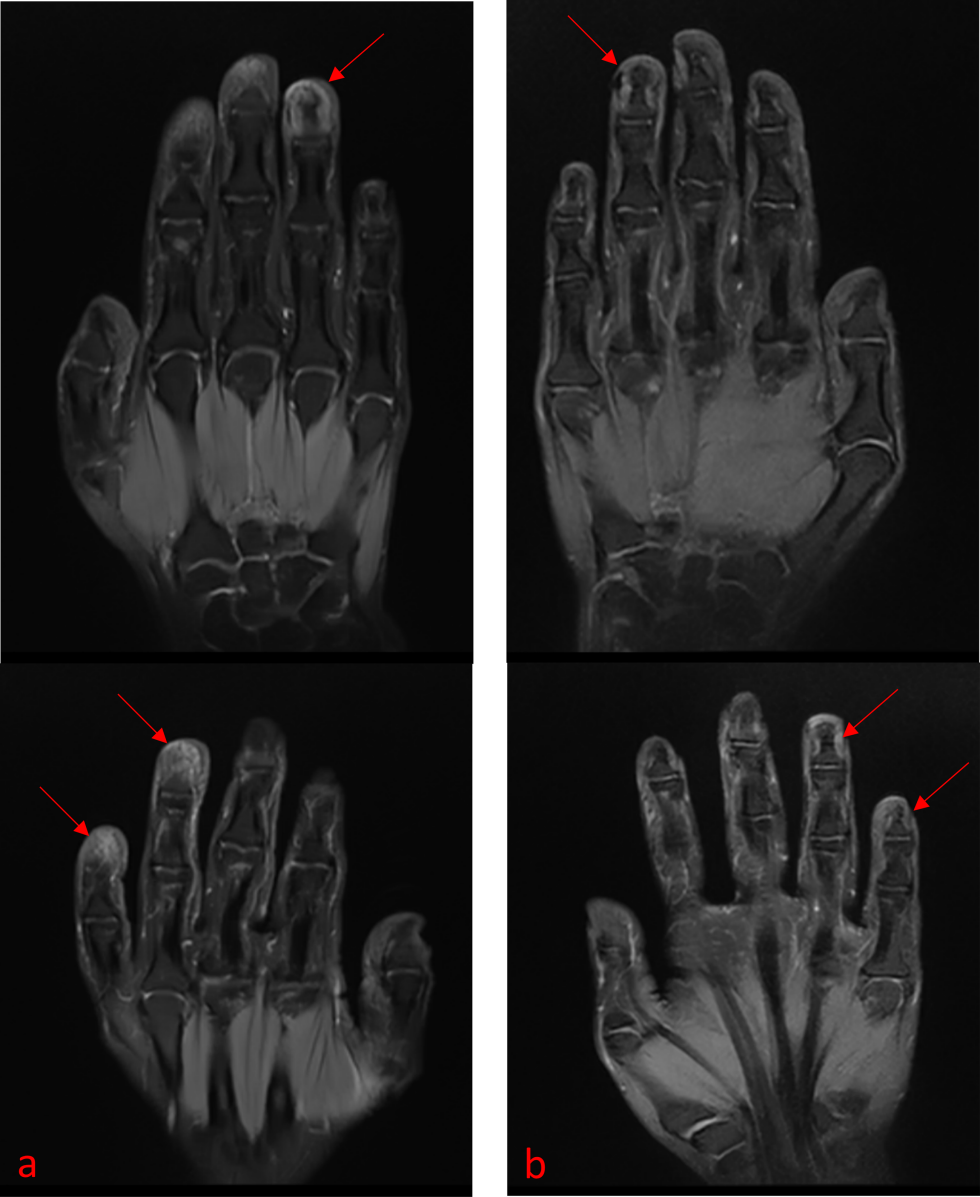
MRI findings at baseline and after treatment with Xeligekimab. The post-treatment COR T2WI-FS sequence indicated marked improvement and partial resolution of the bone-marrow edema in the distal phalanges of the left fourth and right fourth and fifth fingers.

Dermatological examination revealed persistent periungual and subungual erythema and pustules involving the distal phalanges of the left ring finger and right thumb. The nail plates appeared thickened, opaque, longitudinally ridged, and fragmented, with marked dystrophy with partial nail loss. The affected digits exhibited diffuse fusiform swelling, marked tenderness, mildly elevated skin temperature, and limited passive flexion and extension (**Figure 1a**). The Dermatology Life Quality Index (DLQI) score was 18/30, while the Visual Analogue Scale (VAS) pain score was 8/10. Given the presence of marked periungual swelling and pain, magnetic resonance imaging (MRI) was performed to exclude local infection or inflammatory osteitis. The MRI findings showed patchy T2/STIR hyperintensity and T1 hypointensity within the distal phalanges of the left ring finger, right ring finger, and right little finger (**Figure 2a**), consistent with active bone marrow edema. As the patient was concerned that histopathological examination could further exacerbate tissue injury and therefore explicitly declined the procedure, the diagnosis of ACH was established based on the characteristic clinical features and MRI findings.

The first four treatment sessions involved a conservative dosing approach based on injection protocols for nail psoriasis (4). A five-fold dilution of Xeligekimab (100 mg/mL) was prepared by mixing 0.2 mL of the drug with 0.8 mL of normal saline, and 0.1 mL of the diluted solution was injected periungually into both affected digits at two-week intervals. By week 4, the pustules and erythema appeared somewhat reduced, although there was no improvement in the nail growth. By week 8, there were no new pustules, the periungual inflammation had subsided, and the digital swelling was slightly reduced; meanwhile, the DLQI score decreased to 14/30 and the VAS pain score dropped to 5/10.

Starting from the fifth treatment session, undiluted Xeligekimab (0.1 mL) was injected directly into each affected fingertip at two-week intervals, for a total of 10 sessions. By week 12, marked reductions in nail thickening and fragmentation were observed, and healthy nail regrowth was evident. By week 20, the periungual inflammation had resolved completely, with only mild longitudinal ridging visible on the smooth new nail plates, while the digital swelling and tenderness were also fully resolved (**Figure 1b**). The DLQI score had improved to 2/30, and the VAS pain score had decreased to 0/10. A follow-up MRI showed substantially reduced T2/STIR hyperintensity at the previously affected sites of the distal phalanges (**Figure 2b**), indicating significant resolution of the bone marrow edema and near-complete remission of the soft tissue swelling. No local adverse reactions, such as pain, necrosis, infection, or neuropathy, nor any other adverse events, were observed during the course of treatment and follow-up period.

## Discussion

This report is the first description of the successful treatment of refractory ACH using periungual injection of Xeligekimab. ACH, a rare variant of pustular psoriasis, is characterized by the presence of recurrent sterile pustules that can cause severe pain and irreversible destruction of the nails. Xeligekimab is a fully human monoclonal antibody that targets interleukin-17A (IL-17A). The antibody binds selectively to IL-17A, preventing its interaction with the IL-17A receptor complex and the consequent downstream release of pro-inflammatory cytokines and chemokines, thereby attenuating inflammation (5). Its efficacy and safety have been well-established in patients with moderate-to-severe plaque psoriasis and generalized pustular psoriasis (6, 7). A review of the literature identified 12 reported cases of ACH treated with IL-17 inhibitors (**Table 1**), including seven treated with Secukinumab (8–14), two with Ixekizumab (15, 16) and three with Brodalumab (17–19). The treatment durations ranged from 1 to 53 months. Collectively, these studies indicated that IL-17 blockade, either as monotherapy or in combination regimens, was effective in alleviating ACH. However, previous treatments have mostly used systemic administration of biologic agents at standard doses. Compared with the use of systemic biologics at standard doses, local injection of biologic agents is more targeted, faster, and safer (4, 20–22). Local periungual injection may provide more effective antibody delivery to the target site through direct diffusion and interstitial fluid movement, thereby avoiding the need for high systemic concentrations and reducing both costs and adverse effects (20).

**Table 1:** Literature reports of patients with ACH treated with IL-17 inhibitors.

Authors	Age (Years)/Sex	Biologic drug	Combination drugs	Dosage schedule	Outcome
Galluzzo M et al (8)	27/Female	Secukinumab	/	52 weeks of treatment; 300 mg by subcutaneous injection at weeks 0, 1, 2, 3, 4 and every 4 weeks thereafter	Success
Muggli D et al (10)	87/Male	Secukinumab	/	6 weeks of treatment; 300 mg by subcutaneous injection at weeks 0, 1, 2, 3, 4 and every 4 weeks thereafter	Success
Khosravi-Hafshejani T et al (11)	53/Male	Secukinumab	/	2 years of treatment; unknown	Success
Balestri R et al (12)	43/Male	Secukinumab	Acitretin for 4 weeks	10 months of treatment; 300 mg by subcutaneous injection at weeks 0, 1, 2, 3, 4 and every 4 weeks thereafter	Success
Baron JA (13)	42/Female	Secukinumab	/	1 month of treatment; 300 mg by subcutaneous injection at weeks 0, 1, 2, 3, 4	Success
Yao XY et al (14)	44/Male	Secukinumab	Apremilast 60mg/day after 2 years of Secukinumab treatment	4 years and 5 months of treatment; 300 mg by subcutaneously every 4 weeks for 2 years, then (post-combination therapy) every 4 weeks for 5 months and every 8 weeks thereafter	Success
Miller AC et al (15)	31/Female	ixekizumab	/	3 months of treatment; 80 mg by subcutaneous injection every 2 weeks	Success
Battista T et al (16)	72/Male	ixekizumab	/	7 months of treatment; 160 mg by subcutaneous injection at weeks 0 and 80 mg every 2 weeks thereafter	Success
Milani-Nejad N et al (17)	60/Male	Brodalumab	/	6 months of treatment; 210 mg by subcutaneous injection at weeks 0, 1, 2 and every 2 weeks thereafter	Success
Passante M et al (18)	37/Female	Brodalumab	/	6 months of treatment; 210 mg by subcutaneous injection at weeks 0, 1, 2 and every 2 weeks thereafter	Success
Bardazzi F et al (19)	43/Male	Brodalumab	/	12 months of treatment; 210 mg by subcutaneous injection at weeks 1, 2, 3 and every 2 weeks thereafter	Success

A study by He et al. describes the treatment of patients with nail psoriasis with intralesional injections of Secukinumab at concentrations of 7.5, 15, and 30 mg/mL for 12 weeks. All three concentrations resulted in significant clinical improvement, and the sustained local accumulation of the drug extended the duration of the prolonged the post-treatment benefit (23). In the present case, the initial four injections represented a diluted formulation (20 mg/mL), leading to reductions of 22.2% and 37.5% in the DLQI and VAS score, respectively, by week 8. Subsequent treatment with undiluted Xeligekimab resulted in further improvement, with decreases in the DLQI and VAS scores of 85.7% and 100%, respectively, by week 20 compared with week 8. These findings suggest that for complex, treatment-refractory ACH, higher local drug concentrations may induce more rapid and durable responses; however, optimal dosing and safety thresholds require further investigation in controlled studies.

ACH is often accompanied by various changes in bone, including hyperostosis, bone resorption, erosion, and secondary osteomyelitis. Excessive activation of inflammatory mediators may also induce arthritis and bone-marrow alterations. Chronic cutaneous inflammation can extend into adjacent bone tissues, where inflammatory cytokines can disrupt vascular permeability and induce exudation, mediated by hematogenous or direct diffusion pathways. Moreover, ACH may impair the regulation of local neurovascular functions, leading to vasomotor dysfunction and venous stasis that can further exacerbate the bone-marrow edema (24). Although certain small, lipophilic topical agents can penetrate the superficial dermis, even advanced transdermal delivery systems, such as nano-carriers or penetration enhancers, are rarely associated with effective drug concentrations beyond the dermal layer, limiting their efficacy in counteracting deep inflammatory processes such as bone-marrow edema (25). In the present case, the MRI findings revealed marked edema of the bone marrow in the distal phalanges, consistent with inflammatory osteitis. Following periungual injection of Xeligekimab, the bone-marrow edema resolved substantially, indicating that localized high-concentration IL-17 blockade is effective in modulating deep inflammatory activity extending from the skin to the bone.

## Conclusion

Periungual injection of Xeligekimab at a concentration of 100 mg/mL may represent one of the potential optimal treatment options for ACH. However, this study has several limitations. First, as a single-patient case report, statistical quantification is difficult, and the findings cannot be generalized to the broader ACH patient population. Second, the lack of long-term follow-up data precludes assessment of the long-term efficacy and safety of this treatment regimen. In addition, the absence of pharmacokinetic data prevented a precise characterization of the drug distribution profile of Xeligekimab and did not allow further optimization of the dosing strategy. Finally, the potential risks of high concentration periungual biologic injections should not be overlooked, including but not limited to infection, bleeding, nerve injury, and pain, as well as safety issues inherent to the biologic agents themselves.

## Data Availability

The datasets presented in this study can be found in online repositories. The names of the repository/repositories and accession number(s) can be found in the article/supplementary material.
